# Editorial: Nanomedicine in Cancer Targeting and Therapy

**DOI:** 10.3389/fonc.2021.788210

**Published:** 2021-10-25

**Authors:** João Paulo Figueiró Longo, Luis Alexandre Muehlmann, Marcelo Calderón, Christian Stockmann, Ricardo Bentes Azevedo

**Affiliations:** ^1^ Department of Genetics and Morphology, Institute of Biological Sciences, University of Brasília, Brasilia, Brazil; ^2^ Faculty of Ceilândia, University of Brasilia, Brasilia, Brazil; ^3^ POLYMAT, Applied Chemistry Department, Faculty of Chemistry, University of the Basque Country, Universidad del País Vasco/ Euskal Herriko Unibertsitatea (UPV/EHU), Donostia-San Sebastián, Spain; ^4^ IKERBASQUE, Basque Foundation for Science, Bilbao, Spain; ^5^ Institute of Anatomy, University of Zurich, Zurich, Switzerland

**Keywords:** nanomedicine, nanotecehnology, immunology, oncology, innovation

Nanomedicine is a scientific field that uses nanotechnology in the development of diagnostic and therapeutic solutions for medical purposes. The field emerged in the literature during the 1980s, when the first papers involving nanomedical applications were published (1, 2). A second important milestone was the launch, in the 1990s, of the first two pharmaceutical nanomedical products, Doxil^®^, and Myocet^®^, liposomes carrying chemotherapeutical drugs. These oncological applications were important in reducing the toxicity and improving the effectiveness of chemotherapy, thus improving the quality of life of thousands of people (3).

In addition, the most relevant recent application of nanomedicine has been in the development of COVID mRNA vaccines, which involved the use of lipid nanoparticles (**Figure 1**). Due to the instability of the RNA sequences, the use of lipid nanoparticles was a crucial step to keep the integrity of the oligonucleotides. It would be impossible to use these vaccines without the protection and stability conferred by these lipid nanoparticles (4). Indeed, due to the importance of the pandemic, and as these vaccines were applied to billions of people, we can say that it was the most impactful use of nanomedicine to date. Furthermore, these technologies have the potential to be used as new therapeutic platforms for other medical conditions, such as cancer and autoimmune disease, because other possibilities for their use are continuously under investigation (5, 6).

**Figure 1:**
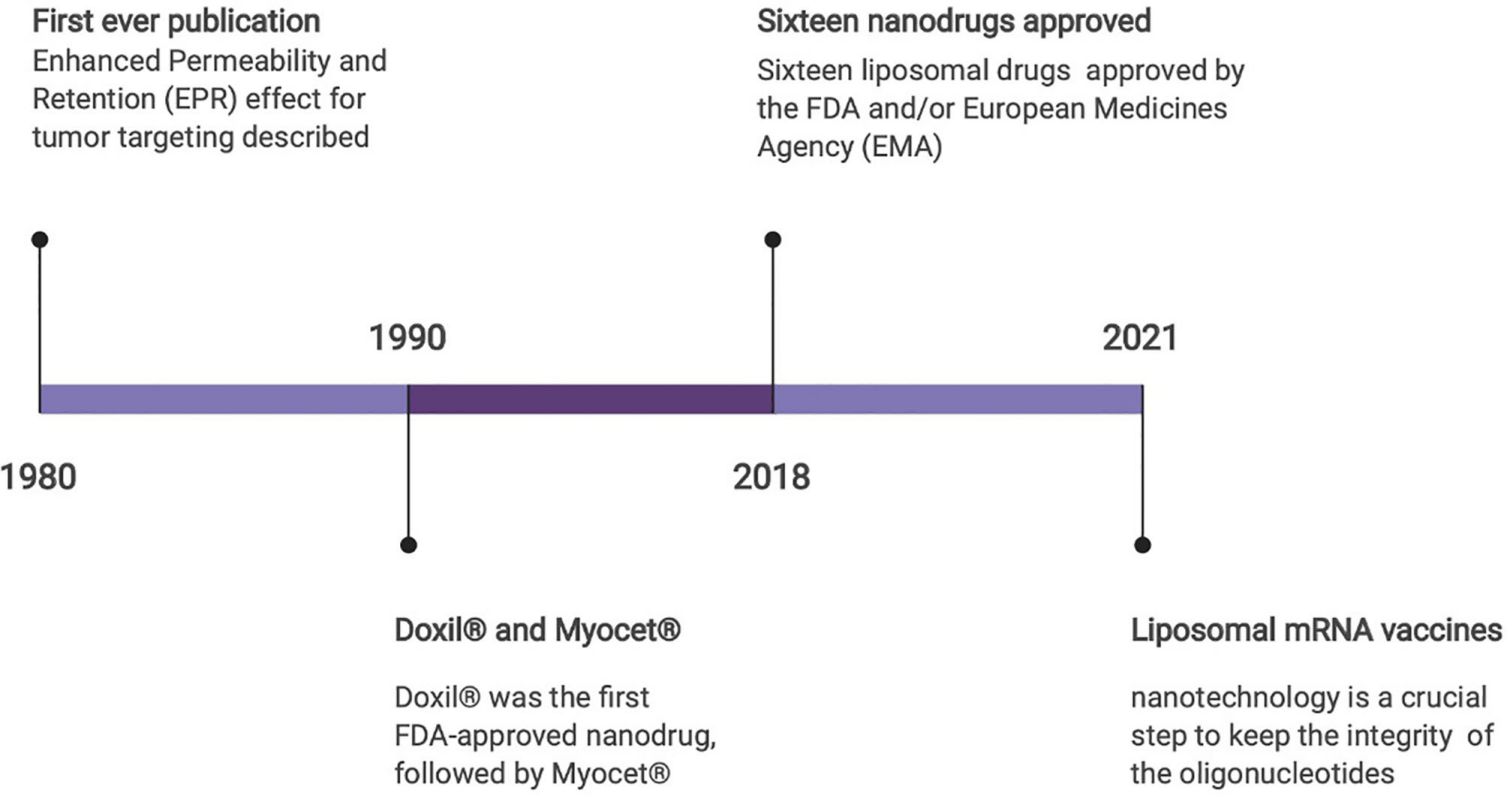
Nanomedicine milestones.

Within this historical context, we proposed this Research Topic to Frontiers in Oncology, aiming to invite authors to publish the most recent scientific and technological advances in the field of nanomedicine. After almost two years, we had received 25 article submissions, and 10 of them were accepted and included in our special issue “Nanomedicine in Cancer Targeting and Therapy”. Five tfmkoriginal articles, six review articles, and one systematic review article were chosen for publication.

Among the original articles, one describes polymeric nanoparticles used to encapsulate gambogic acid, a phytochemical compound commonly used in Chinese medicine. As the main results, Kwan et al. showed the effectiveness of this nanocarrier against triple-negative breast cancer cells, in both *in vitro* and *in vivo* models. Another original article describing a nanoformulation of natural products was published by Xu et al. The authors showed that *Ginkgo biloba* aqueous extract was a useful natural source to synthesize silver nanoparticles by a green method. The authors also showed that the prepared silver nanoparticles were able to induce apoptosis in cervical cancer cells. They suggested that these silver nanoparticles could be used as an alternative therapy for cervical cancer.

Still in the context of natural products, we received the submission of a systematic review published by Ombredane et al. In this article, the authors presented an important overview on the use of curcumin, a plant-derived molecule, in the treatment of breast cancer, and how nanoencapsulation could improve the effectiveness of this compound. This approach is interesting, because several plant-derived compounds have problems such as instability and/or solubility, issues that impair their use in biomedical applications.

Another important application of nanoparticles in medical applications is the ability to modulate the pharmacokinetics of small-molecule drugs. Two review articles discussed the ability of different types of nanocarriers to optimize the effect of two small-molecule drugs, methotrexate and nimesulide. Yu et al. presented some insights regarding the use of nanoparticles as drug delivery agents to target methotrexate for both cancer and rheumatoid arthritis. And Ferreira et al. discussed the use of nanotechnology to improve the efficacy of nimesulide as a repositioned drug for the treatment of pancreatic tumors, due to its anti-inflammatory properties.

The last original article presented here was published by Cui et al. In this report, the authors describe a novel PLGA nanoparticle coated with red blood cell membranes and decorated with two different peptides. The aim of this interesting surface modification was to improve the crossing of the blood-brain barrier, a property that was achieved as shown in mice models for glioma. The strategy of using biological membranes in nanomedicine was also the subject of two review articles available in this edition. These two reviews, published by Ma at al. and Chen et al., described the potential use of extracellular vesicles, or exosomes, as candidate nanosized drug carriers for cancer therapy. These natural vesicles were the inspiration for liposome development, and are basically composed of a phospholipid bilayer containing cholesterol and also proteins, an organization resembling that of cell membranes. As the main advantage in comparison to liposomes, these natural vesicles can overcome natural barriers and can circulate freely in the bloodstream. Moreover, the presence of specific proteins on the vesicle surface can address these exosomes to specific target tissues. Limiting issues regarding the industrial biotechnology production of these natural nanocarriers are also discussed in the two review articles.

Finally, we present two review articles discussing some innovative approaches in the nanomedical field. First, Franco et al. present some important issues related to the tumor environment that could be used to trigger the release of carried drugs in the tumor site. This is an interesting approach that has also been published by our research group. Finally, Javed et al. reviewed some important aspects of gene signaling by miRNA as a potential therapy for colorectal cancer. The instability of nucleic acids requires the use of drug delivery systems, such as nanocarriers, that can protect and transport miRNA to target cells.

In conclusion, this special issue brings together a group of original and review articles addressing different aspects of nanomedicine. We believe that this published information can help to understand nanomedicine more deeply and to develop new therapeutic approaches for cancer. We are certain that all the high-quality information published in this issue is interesting both to researchers and to those interested in alternative cancer treatments using nanotechnological approaches.

## Author Contributions

JL – discuss the editorial content and prepared the first version of the editorial. LM, MC, CS and RA - discuss the editorial content and revised the final editorial text. All authors contributed to the article and approved the submitted version.

## Conflict of Interest

The authors declare that the research was conducted in the absence of any commercial or financial relationships that could be construed as a potential conflict of interest.

## Publisher’s Note

All claims expressed in this article are solely those of the authors and do not necessarily represent those of their affiliated organizations, or those of the publisher, the editors and the reviewers. Any product that may be evaluated in this article, or claim that may be made by its manufacturer, is not guaranteed or endorsed by the publisher.
